# Assessment of the in vitro acaricidal activity of Bravecto^®^ (fluralaner) and a proposed orange oil-based formulation vehicle for the treatment of *Sarcoptes scabiei*

**DOI:** 10.1186/s13071-024-06275-9

**Published:** 2024-04-25

**Authors:** Kotaro Takano, Scott Carver, Yolandi Vermaak, Katja Fischer, Robert J. Harvey, Kate E. Mounsey

**Affiliations:** 1 School of Health, University of the Sunshine Coast, Maroochydore, QLD Australia; 2 Sunshine Coast Health Institute, Birtinya, QLD Australia; 3 Odum School of Ecology, University of Georgia, Georgia, USA; 4 Center for the Ecology of Infectious Diseases, University of Georgia, Georgia, 30602 USA; 5 Wombat Support and Rescue NSW/ACT Inc., Australian Capital Territory, Canberra, Australia; 6 Infection and Inflammation Program, QIMR Berghofer Medical Research Institute, Brisbane, QLD Australia

**Keywords:** *Sarcoptes scabiei*, Sarcoptic mange, Scabies, Bare-nosed wombat, *Vombatus ursinus*, Fluralaner, Citrus oil, Essential oil

## Abstract

**Background:**

Sarcoptic mange is a serious animal welfare concern in bare-nosed wombats (*Vombatus ursinus*). Fluralaner (Bravecto^®^) is a novel acaricide that has recently been utilised for treating mange in wombats. The topical ‘spot-on’ formulation of fluralaner can limit treatment delivery options in situ, but dilution to a volume for ‘pour-on’ delivery is one practicable solution. This study investigated the in vitro acaricidal activity of Bravecto, a proposed essential oil-based diluent (Orange Power^®^), and two of its active constituents, limonene and citral, against *Sarcoptes scabiei*.

**Methods:**

*Sarcoptes scabiei* were sourced from experimentally infested pigs. In vitro assays were performed to determine the lethal concentration (LC_50_) and survival time of the mites when exposed to varying concentrations of the test solutions.

**Results:**

All compounds were highly effective at killing mites in vitro. The LC_50_ values of Bravecto, Orange Power, limonene and citral at 1 h were 14.61 mg/ml, 4.50%, 26.53% and 0.76%, respectively. The median survival times of mites exposed to undiluted Bravecto, Orange Power and their combination were 15, 5 and 10 min, respectively. A pilot survival assay of mites collected from a mange-affected wombat showed survival times of < 10 min when exposed to Bravecto and Orange Power and 20 min when exposed to moxidectin.

**Conclusions:**

These results confirm the acaricidal properties of Bravecto, demonstrate acaricidal properties of Orange Power and support the potential suitability of Orange Power and its active constituents as a diluent for Bravecto. As well as killing mites via direct exposure, Orange Power could potentially enhance the topical delivery of Bravecto to wombats by increasing drug penetration in hyperkeratotic crusts. Further research evaluating the physiochemical properties and modes of action of Orange Power and its constituents as a formulation vehicle would be of value.

**Graphical Abstract:**

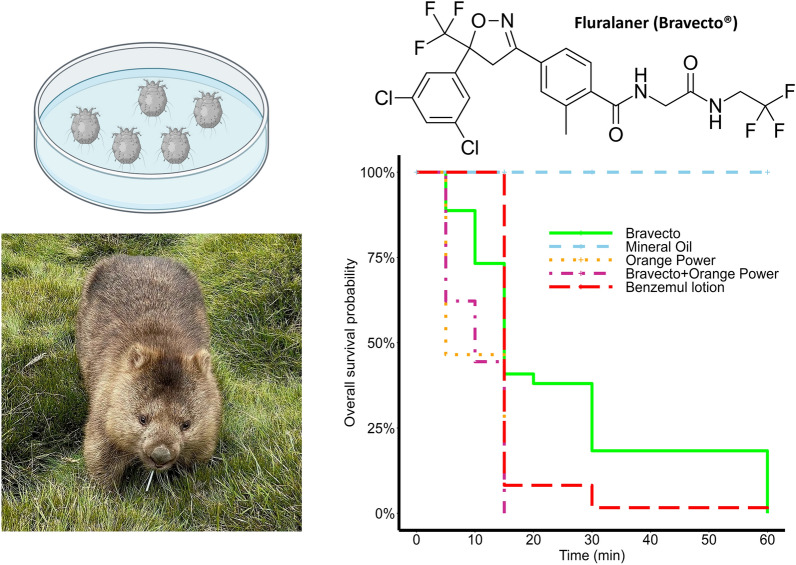

## Background

In Australia, bare-nosed wombats (*Vombatus ursinus*) are highly susceptible to *Sarcoptes scabiei* infestation. Sarcoptic mange represents a serious animal welfare concern in this species, and mange outbreaks have been associated with considerable population declines in some regions. Populations in Narawntapu National Park and Bronte Park in Tasmania experienced 94% and 80% declines, respectively, because of outbreaks of sarcoptic mange [1–3]. Treating sarcoptic mange in free-living populations of wombats is especially challenging, since commonly utilised acaricides, ivermectin and moxidectin, are often insufficient to clear infestation with a single dose because of their short plasma half-life, particularly in wombats (reviewed in Takano et al., 2023 [4]).

Fluralaner (Bravecto^®^; MSD Animal Health), belonging to the isoxazoline class of insecticides, is a promising new treatment for sarcoptic mange, with a notably longer duration of effectiveness compared to ivermectin and moxidectin. The application of topical fluralaner to bare-nosed wombats demonstrated prolonged retention (plasma half-life and mean residence time of 40 and 32 days for 25 mg/kg and 167 and 47 days for 85 mg/kg, respectively) [5]. The clinical efficacy of Bravecto against sarcoptic mange has been documented in several species, including wombats [5–8]. Bravecto has been approved by the Australian Pesticides and Veterinary Medicines Authority (APVMA) for the treatment of sarcoptic mange via direct application. However, the ‘spot-on’ formulation is not always practicable to administer via commonly employed non-invasive methods such as ‘pole and scoop’ or ‘burrow flap’ (reviewed in Wilkinson et al., 2021 [5]) because of its low volume (< 3.57 ml per pipette). Due to the fur and hyperkeratotic plaques present in sarcoptic mange-affected wombats, adequate topical drug delivery is of key importance [9]. Since it may be challenging to deliver a sufficient volume of Bravecto to free-living wombats, increasing the volume of the administered dose by mixing Bravecto with a suitable pour-on formulation could improve delivery and potentially improve penetration and distribution [10]. ‘Orange Power Sticky Spot and Goo Dissolver®’ (Aware Environmental Products/Hiro Brands, VIC, Australia; hereafter Orange Power), containing orange peel and other citrus essential oils, has been proposed as a candidate vehicle for the spot-on formulation of Bravecto. Advantages include ready retail availability of Orange Power and constituents, cost effectiveness and compatible solvent properties for Bravecto [5]. Orange Power and its constituents also have high volatility so likely evaporate from the host relatively rapidly after exposure. Additionally, it is well established that many plant essential oils and their constituents such as terpenes have demonstrated acaricidal activity, including against *S. scabiei* [11]. However, the acaricidal effect of Orange Power and key constituents have not yet been evaluated in *S. scabiei*.

This study aimed to evaluate the in vitro efficacy of Bravecto, Orange Power, limonene and citral on *S. scabiei*. Since no study to our knowledge has documented in vitro activity of Bravecto on *S. scabiei*, this will contribute to defining a baseline drug response phenotype in a population of mites previously unexposed to acaricides as well as provide further information on the suitability of Orange Power and its constituents as a vehicle for the spot-on Bravecto formulation to treat free-living animals including wombats.

## Materials

### Mites and compounds

*Sarcoptes scabiei* were sourced from skin crusts collected from the ears of experimentally infested pigs housed at the Queensland Animal Science Precinct (University of Queensland, Gatton, Australia). Ethical approval was obtained from the QIMR Berghofer Medical Research Institute and University of Queensland animal ethics committees (P630/2021/AE001015). The establishment and protocols for this experimental model have been described elsewhere [12]. These mite populations have had no previous acaricide exposure. Skin scrapings were transported to the laboratory within 2 h, placed on glass petri dishes and incubated at 37 °C for 30 min to isolate mites by encouraging them to crawl from the crusts towards the heat source.

In vitro drug sensitivity assays were conducted using Bravecto^®^ Spot-On for Large Dogs (280 mg/ml fluralaner, MSD Animal Health), Orange Power Sticky Spot and Goo Dissolver^®^ (Aware Environmental Products/Hiro Brands, VIC, Australia) (Table 1) and two Orange Power constituents, limonene (97% purity, Sigma-Aldrich) and citral (geranial, 95% purity, Sigma-Aldrich). Limonene is present as the majority monoterpene in Orange Power, and citral, despite being present in lower proportions, was also included because it belongs to a different chemical class (acyclic monoterpene aldehyde versus cyclic monoterpene) and has previously reported acaricidal activity [13]. Mineral oil (Sigma Aldrich) was used as a negative control/diluent and 25% benzyl benzoate (Benzemul lotion, McGloins Pty Limited, Bella Vista, NSW, Australia) was used as a positive acaricide control. In the pilot assay on wombat-derived *S. scabiei*, Cydectin^®^ Pour-on for Cattle (5 g/l moxidectin, Virbac) was also included because of the frequent utilisation of this formulation in wombat mange treatment.

**Table 1:** Orange Power (Aware Environmental Products/Hiro Brands) and Bravecto Spot-On (MSD Animal Health) ingredients

	Orange Power	%	Bravecto Spot-on	%
Ingredients	Ethanol	30–60	Fluralaner	28
	D-limonene	30–60	Dimethylacetamide	30–40
	Citrus terpenes	< 10	Diethyltoluamide	10–20
	Glycol ether	10–60	Acetone	10–20
	Alcohols ethoxylated	< 10		

### In vitro assays

In vitro assays commenced within 4 h of mite collection. The experiments were divided into two objectives: (1) determining the lethal concentration (LC_50_) of the test compounds against *S. scabiei* and (2) determining the median survival time of *S. scabiei* in response to the test compounds individually and in combination. For experiment 1, test compounds were diluted twofold in mineral oil to obtain test concentrations in the range of 0.44—280 mg/ml fluralaner for Bravecto, 0.78—100% for Orange Power, 0.78—50% for limonene and 0.19—50% for citral; 0.5 ml test solutions and positive or negative controls were added to six-well 30-mm cell culture plates. For experiment 2, undiluted Bravecto and Orange Power were tested individually and in combination. For the combination, 2 ml Bravecto was diluted into 20 ml Orange Power as this represents the approximate ratio used for application in pilot studies on wombats in the field [5].

For experiments 1 and 2, 10 adult female mites were added to each test well. Mite mortality was defined as lack of movement when touched with a probe. For LC_50_ assays, mortality was recorded at 15 min, 30 min, 1 h, 3 h, 5 h and 24 h post-exposure. For survival analysis, mortality was recorded at 5-min intervals to 30 min, 60 min, 3 h, 5 h and 24 h post-exposure. In all assays, the petri dishes were incubated at room temperature and 75% relative humidity. Assays were performed in duplicate and repeated at least three times on separate occasions.

In a pilot survival assay (experiment 3) to test the activity of compounds on mites collected from a mange-infested wombat, collection and assay methods were adapted to a “field-friendly” protocol, requiring minimal technical expertise and time. Briefly, a small amount of skin crust was placed directly into the six-well culture plates and incubated at 37 °C for 10 min to allow mites of all life stages to crawl out of the crusts. The skin crusts were removed, and 0.5 ml diluted test compound was applied. Compounds included mineral oil (negative control), Bravecto (20 mg/ml), Orange Power (10%) and moxidectin (50 µg/ml). Dilutions were selected based on the LC_50_ data and previous data for moxidectin [14, 15], with the expectation that drug-susceptible mites should die within 1 h of exposure to these concentrations. Mites were observed every 5 min for the first 30 min and hourly thereafter for 2 h.

### Statistical analysis

All statistical analysis was performed using R. For experiment 1, LC_50_ values at 1, 3, 5 and 24-h post-exposure were obtained by normalised dose-response analysis and the four-parameter log-logistic function using the ‘drm’ function in the ‘drc’ package [16]. Standard deviations were calculated using the ‘sd’ function. Dose-response curves were plotted by nonlinear regression using the ‘drc’ and ‘ggplot2’ packages.

For experiments 2 and 3, Kaplan-Meier survival curves and their 95% confidence intervals were visualised using the ‘survfit’ and ‘ggsurvplot’ functions in R packages ‘survival’ [17, 18] and ‘ggsurvfit’ [19]. A pairwise comparison of the survival curves between the test solutions was conducted using log-rank test in the ‘pairwise_survdiff’ function in the ‘survminer’ package [20].

## Results

### LC_50_ assays

**Fig. 1:**
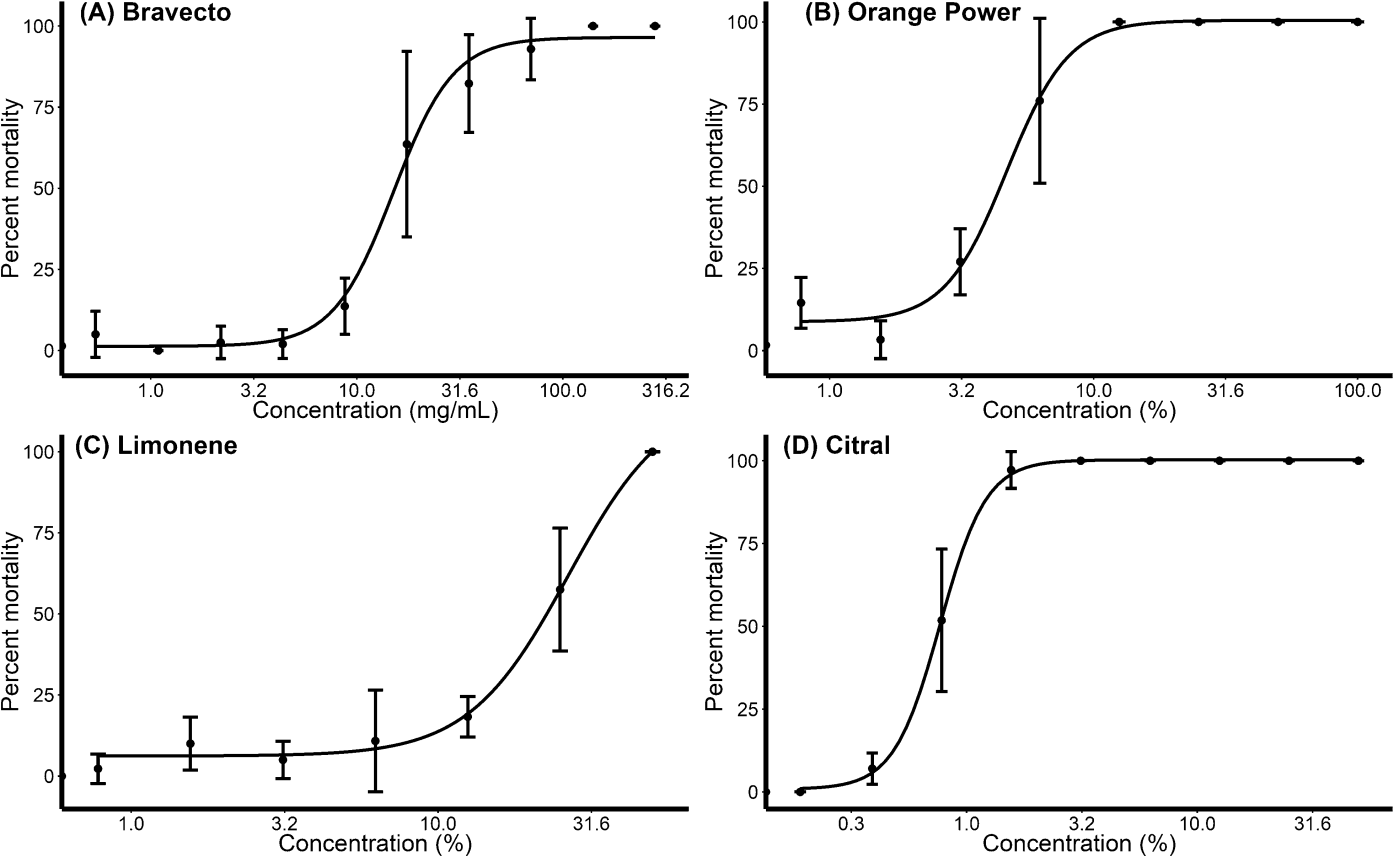
Dose-response curves of *Sarcoptes scabiei* mortality to Bravecto, Orange Power, limonene and citral. Mites were exposed for 1 h in vitro to serial dilutions of **A** 0 to 280 mg/ml Bravecto, **B** 0 to 100% Orange Power, **C** 0 to 50% limonene and **D** 0 to 50% citral. Points show median mortality; bars show standard deviations

At 1 h post-exposure, the LC_50_ values of Bravecto, Orange Power, limonene and citral were 14.61 mg/ml, 4.50%, 26.53% and 0.76%, respectively (Table 2; Fig. 1). LC_50_ values decreased at 3 h for all compounds, and at 24 h the LC_50_ values for Bravecto and limonene had decreased to 2.17 mg/ml and 4.56%, respectively (Table 2). The LC_50_ values of Orange Power and citral at 24 h could not be obtained because of 100% mortality rates at all concentrations (Table 2; Fig. 1).

**Table 2:** LC_50_ values for Bravecto, Orange Power, limonene and citral for scabies mites

Chemical	1 h		3 h		5 h		24 h	
	LC_50_	95% CI	LC_50_	95% CI	LC_50_	95% CI	LC_50_	95% CI
Bravecto (mg/ml) (*n* = 496)	14.61	12.20–17.01	7.62	5.83–8.86	4.94	2.89–6.98	2.17	1.76–2.58
Orange power (%) (*n* = 367)	4.50	3.85–5.14	3.11	2.3–3.91	2.11	1.59–2.63	–	–
Limonene (%) (*n* = 284)	26.53	16.43–36.63	8.42	5.31–11.54	7.14	2.84–11.46	4.56	3.42–5.70
Citral (%) (*n* = 372)	0.76	0.70–0.83	0.65	0.51–0.78	0.63	0.49–0.78	–	–

*LC*_*50*_ lethal concentration 50, amount required to cause death in 50% of mites

*95% CI* 95% confidence interval

### Survival analysis

**Fig. 2:**
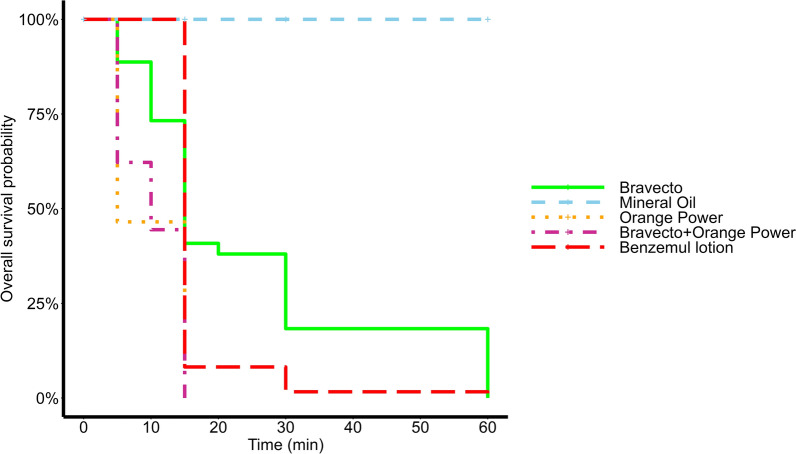
Kaplan-Meier survival curves of *Sarcoptes scabiei* exposed to Bravecto, Orange Power or a Bravecto/Orange Power combination. Mites were exposed to Bravecto (*n* = 71), Orange Power (*n* = 43) or the combination of 2 ml Bravecto and 20 ml Orange Power (*n* = 45). Benzemul lotion (*n* = 61) and mineral oil (*n* = 61) were used as positive and negative controls

Survival analysis showed that the median survival time of the negative control (mineral oil) was > 24 h, while the median survival time of the positive control (Benzemul lotion) was 15 min (Fig. 2). All test solutions demonstrated significant acaricidal activity compared to the negative control (*p* < 0.001). The median survival times of mites exposed to 100% Bravecto, Orange Power and the combination treatment were 15 min, 5 min and 10 min, respectively (Fig. 2). Pairwise comparisons using log-rank test revealed significant differences in median survival times between Bravecto and mineral oil (*p* < 0.001), Bravecto and Orange Power (*p* < 0.001) and Bravecto and the combination treatment (*p* < 0.001). Significant differences were also found between mineral oil and Benzemul lotion (*p* < 0.001), mineral oil and Orange Power (*p* < 0.001), mineral oil and the combination (*p* < 0.001), Orange Power and Benzemul lotion (*p* < 0.001) and the combination and Benzemul lotion (*p* < 0.001). No significant differences in median survival time were observed between Orange Power and the combination (*p* > 0.999) and between Bravecto and Benzemul lotion (*p* > 0.999).

In the pilot assay to test drug activity on wombat mites of mixed life stages in the field, Bravecto, Orange Power and Cydectin were tested and compared to the mineral oil-only negative control. Mite numbers between treatments varied because of differences in the numbers of mites contained in crusts. The results for Bravecto and Orange Power were consistent with the data from pig-derived mites, with Orange Power the fastest acting (mites dead within 5 min), followed by Bravecto (mites dead within 10 min) and then Cydectin (mites dead within 20 min) (Table 3). Mites remained alive in mineral oil for the 2-h observation period.

**Table 3:** Pilot survival assay on *Sarcoptes scabiei* collected from a mange-infested wombat

Test solution	Concentration	n	Median survival time (min)
Orange Power	10%	10	5
Bravecto (fluralaner)	20 mg/ml	25	10
Cydectin (moxidectin)	50 µg/ml	100	20
Mineral oil (negative control)		20	> 120

## Discussion

This study assessed the in vitro activity of Bravecto and Orange Power and constituents to provide information on their baseline drug response phenotypes in *S. scabiei*. While Bravecto has demonstrated clinical efficacy against sarcoptic mange, no data were previously available on in vitro activity. Orange Power has attracted recent interest as a potential formulation vehicle in which to dilute Bravecto and facilitate delivery as a pour-on treatment of sarcoptic mange in wombats [5]. Bravecto, Orange Power and constituents showed rapid in vitro acaricidal activity at low concentrations. Mites died within 5 min of exposure to undiluted Orange Power and within 15 min of exposure to Bravecto. The mixture of Bravecto with Orange Power, as used in field application, resulted in a median survival of 10 min, confirming that there were no antagonistic effects of combining these products while demonstrating significantly higher in vitro efficacy compared to Bravecto alone.

Scabies mites obtained opportunistically from a mange-infested wombat were also tested, confirming the potent acaricidal activity of Bravecto and Orange Power and the applicability of these results to different host-derived variants of *S. scabiei*. Survival times for wombat-derived mites were similar, although lower concentrations were tested, and mites also died rapidly upon exposure to moxidectin. The wombat-mite assays did not select for female-only mites, and a larger number of juvenile mites (nymphs and larvae) were tested in these assays. Previous testing of moxidectin has shown that survival times are significantly reduced in juvenile stages relative to adult females [14], which is in accordance with our current results. This pilot assay, where methods were adapted for ease of testing, may be useful for ongoing monitoring of efficacy in wombat mange treatment programs.

The observed in vitro efficacy of Bravecto is in accordance with known demonstrated clinical efficacy [5]. Fluralaner has not been tested previously against *S. scabiei* in vitro, although the related isoxazolines, afoxolaner and sarolaner, were tested at a concentration of 25 mg/ml, which resulted in approximately 90 and 70% mortality respectively after 2 days [21]. Direct comparison with these results with our study is limited by the fact that we could not test pure fluralaner because of inadequate solubility at the test concentrations required. Other acari species that are highly susceptible to in vitro fluralaner exposure include the brown dog tick (*Rhipicephalus sanguineus*), African hut tampan (*Ornithodoros moubata*) [22] and poultry red mite (*Dermanyssus gallinae*) [23]. The LC_50_ concentrations obtained in these studies were significantly lower than those for *S. scabiei* in our study. These differences may be due to differences in observation times (minutes/hours vs. days) and differences in vitro methods such as direct feeding, contact or fumigant exposure. Since *S. scabiei* is an obligate skin-feeding ectoparasite, it cannot be sustained in a culture environment. Thus, the assays conducted in the present study are likely to represent the activity through contact exposure only rather than the combination of contact exposure and ingestion of acaricides that would occur in an in vivo setting. It has been previously documented that there is a discrepancy between drug concentrations required for killing mites in vitro and the effective drug concentrations used in vivo for *Sarcoptes* and other parasites [14]. Therefore, it is crucial to acknowledge that these assays represent relative, not absolute activity [24].

Many plant-derived essential oils, especially terpenoids, have demonstrated efficacy against *S. scabiei* [11]. However, prior to this study, acaricidal activity of Orange Power had not been demonstrated in vitro or in vivo. Our results are in accordance with other studies on related citrus oils. For example, exposure of *S. scabiei* to 10% bitter orange oil (*Citrus aurantium amara*) resulted in a median survival time of 20 min [25]. In comparison, lemon oil (*Citrus limon*) was associated with longer lethal exposure times (10%, 72% mortality at 1 h post-exposure) [26]. More recently, exposure of mites to 20% mandarin peel oil resulted in 99% mortality at 24 h post-exposure [27].

While the citrus oils tested above have similar constituents to Orange Power, it is important to recognise that they often consist of complex mixtures with main constituents including terpenes. Many, including Orange Power, have limonene as the main component. Other common constituents present in orange oils in lower proportions include α-phellandrene, α-thujene, linalool, β-pinene, myrcene and citral [26, 28]. To further study the activity of orange oil, we also tested two main constituents—limonene and citral (also referred to as geranial or trans-citral). In our results, citral had higher activity (LC_50_ at 1 h post-exposure of 0.76%) compared to limonene (LC_50_ at 1 h post-exposure of 26.53%). While the in vitro efficacy of limonene has not been directly tested in *S. scabiei* before, strong activity of citral has been documented, with exposure to 5% resulting in 100% mortality within 25 min post-exposure [13]. In addition, 1% citral resulted in 100% mortality at 30 min post-exposure in a closely related species, *Psoroptes cuniculi* [29].

One key mechanism of the acaricidal activity of citrus oils may be cellular damage via membrane disruption. Aboelhadid et al. (2016) observed a high level of hydrogen peroxide and lipid peroxide activity in *S. scabiei* exposed to 20% lemon oil, suggesting reactive oxygen species induced by limonene resulted in oxidation and breakdown of phospholipids and fatty acids in the cell membrane and led to cellular apoptosis and/or swelling in the mites [26, 30, 31]. This cellular damage can manifest in mites and insects as damage to the fat body, cuticle, epidermis, digestive tract and brain, leading to death [32].

Various essential oil components, including limonene and citral, have also been reported to act as acetylcholinesterase (AChE) inhibitors. This represents a common target of other insecticides, including carbamates and organophosphates [33]. In *Rhipicephalus microplus*, AChE IC_50_ (50% inhibitory concentration) values of limonene and citral were 1.63 and > 5.0 mg/ml, respectively [34]. Limonene was reported to be a weak inhibitor of AChE from the two-spotted spider mite (*Tetranychus urticae*), with an IC_50_ value of 12.48 mg/ml after 30 min post-exposure [35]. In contrast, Oyedeji et al. (2020) observed that limonene and citral exhibited robust inhibitory activity against AChE from the maize weevil (*Sitophilus zeamais*) and the cowpea weevil (*Callosobruchus maculatus*), reducing its activity by 40–50% after 2 min exposure at the concentrations of their LC_50_ against these species [36]. Although less established as target sites of essential oils, ligand-gated chloride channels (LGICs) including γ-aminobutyric acid (GABA) receptors have been reported to interact with thymol, carvacrol and pulegone, which reversibly bind to insect and mammalian GABA receptors and increase chloride flux by acting as positive allosteric modulators [37–39]. These results provide insights on the possible varying modes of action of citral and limonene in this context although further research on detailed modes of action of limonene and citral in *S. scabiei* is required. Diversity in target activity by essential oil-based products could be a significant advantage in combatting drug resistance, which often involves genetic variations in single target sites such as the LGICs.

Our assays focused only on adult mites, although it is acknowledged that treatments targeting all life stages, including eggs, would be highly beneficial to the management of sarcoptic mange [21]. Previous in vitro assays on Afoxolaner, another isoxazoline compound, suggested a lack of ovicidal activity [40]. This may be attributed to eggs lacking a developed nervous system. While there are no studies on the ovicidal activity of limonene against *S. scabiei*, Li et al. (2021) found that 12 h in vitro exposure to 5% linalool, terpinen-4-ol, citral and eugenol resulted in egg killing rates of 36.7%, 48.3%, 50.0% and 100%, respectively [13]. This suggests that these terpenes may penetrate *S. scabiei* eggshells with ovicidal effects to varying degrees and that Orange Power may have ovicidal properties that warrant further testing.

Studies on formulation vehicles for topical veterinary medicines are limited. Using essential oil-based products as formulation vehicles could potentially increase transdermal penetration and distribution [41, 42]. For example, using tea tree oil as a carrier for crotamiton resulted in greater skin penetration and distribution in a microemulsion hydrogel formulation, with no impacts on skin histology [10]. Tea tree oil (*Melaleuca alternifolia*) has demonstrated acaricidal activity and is commonly incorporated into benzyl benzoate lotions to increase tolerability in the treatment of crusted scabies [43]. Furthermore, citrus-derived essential oils are known to possess anti-inflammatory properties [44]. As recovery of skin and resolution of symptoms can be slow because of the ongoing allergic and inflammatory response to mite products, products that kill mites while supporting symptomatic relief would be ideal. This has been demonstrated in a recent in vivo study on rabbits experimentally infested with *S. scabiei*, where a topical administration of mandarin peel (*Citrus reticulata*) essential oil resulted in decreased inflammatory infiltrate with significant improvement of the epidermis [27].

The use of essential oils in the treatment of scabies is a popular topic, and while many have shown promising in vitro activity, few studies have demonstrated clinical efficacy relative to other ‘conventional’ acaricides. While our results support that Orange Power has direct acaricidal activity and may support the delivery of Bravecto, we caution against the interpretation that Orange Power alone would be a suitable ‘alternative’ treatment. First, while mites and possibly eggs may be killed upon contact at the application site, small terpenes including limonene are highly susceptible to degradation and evaporation through isomerisation and oxidation [45], so prolonged activity would not be anticipated. Notably, essential oils can cause skin irritation and/or sensitisation in some cases. Mice exposed to limonene daily for 7 days exhibited histological changes to the skin including hyperkeratinised stratum corneum and ulcerative eruptions [46]. In patch testing of 3.5% citral, 9% patients exhibited skin irritation consistent with allergic contact dermatitis [47]. While Orange Power has demonstrated safety and efficacy in pilot studies on sarcoptic mange in captive wombats (Wilkinson et al., 2023, submitted), and the low frequency of treatment support suggests a satisfactory safety profile [5], further research is warranted, particularly in relation to pre-existing skin irritation. Dilution with Orange Power is most relevant for pour-on using the ‘pole and scoop’ delivery method, because the cost of Bravecto often precludes multiple simultaneous deployments using ‘burrow flaps’. Nonetheless, use of ‘burrow flaps’ is still plausible, and research evaluating whether the odour of Orange Power (or other agents, such as Cydectin and Bravecto) influences how wombats interact with burrow flaps and treatment delivery success, given their strong olfactory senses [48, 49], is warranted.

## Conclusions

Bravecto is efficacious against *S. scabiei* and dilution with Orange Power may support delivery as a pour-on to treat sarcoptic mange in free-living wombats. Our in vitro study showed that Orange Power, and the combination of Orange Power with Bravecto had an equal or higher acaricidal effect and efficacy against *S. scabiei* compared to Bravecto alone, since both compounds exhibit acaricidal activity. Essential oils have potential to enhance efficacy of acaricide penetration and distribution in the host, and their volatility may also be beneficial for minimising host exposure. Further research on ovicidal activity, modes of action and effects of formulation vehicles in vivo would be of value.

## Data Availability

All data generated or analysed are included in this published article.
